# Variant Bilateral Foramina of the Middle Cranial Fossa

**DOI:** 10.7759/cureus.33014

**Published:** 2022-12-27

**Authors:** Arman Raz, Łukasz Olewnik, Georgi P Georgiev, Joe Iwanaga, R. Shane Tubbs

**Affiliations:** 1 Neurosurgery, Tulane University School of Medicine, New Orleans, USA; 2 Department of Anatomical Dissection and Donation, Medical University of Lodz, Lodz, POL; 3 Orthopaedics and Traumatology, University Hospital Queen Giovanna - ISUL, Sofia, BGR

**Keywords:** cadaver, foramen spinosum, foramen rotundum, foramen ovale, middle cranial fossa

## Abstract

Variations of the foramina located at the skull base can have direct clinical implications. For example, transcutaneous approaches to the trigeminal nerve using long spinal needles for treatment of trigeminal neuralgia can inadvertently enter such variant foramina and potentially result in hemorrhage. Therefore, knowledge of such variant foramina is important to the clinician treating or diagnosing patients based on imaging of this region. We report an adult male skull found to have unusual foramina located at the skull base. The foramina were located approximately 3.1 cm lateral to the plane of the foramen rotundum and foramen ovale. The left foramen had a diameter of 0.82 mm and the right foramen had a diameter of 0.77 mm. Both foramina opened up just medial to the sphenotemporal suture into the roof of the infratemporal fossa. Additionally, each foramen was the most lateral of a larger collection of foramina found to exit the skull base but confluent with the diploic space of the greater wind of the sphenoid and not with the floor of the middle cranial fossa. This group of openings, including the most lateral which communicated with the middle cranial fossa, were lateral to the lateral plate of the pterygoid process. Knowledge of variant foramina of the skull base is important to clinicians treating patients with pathology of this region. To our knowledge, foramina as described herein have not been previously reported in the extant medical literature.

## Introduction

The middle cranial fossa is the region of the basilar skull composed anteriorly by the greater wings and chiasmatic sulcus of the sphenoid bone and posteriorly by the petrous portion of the temporal bone. Within the middle cranial fossa lies a complex labyrinth of important osteological structures including foramina for several cranial nerves involved in eye movement, as well as pathways for crucial blood vessels such as the internal carotid artery [1]. The superior orbital fissure is located posterolateral to the chiasmatic sulcus of the sphenoid bone between the greater and lesser wings. The oculomotor nerve (CN III), trochlear nerve (CN IV), ophthalmic branch of the trigeminal nerve (CN V1), and abducens nerve (CN VI) exit through this fissure. The foramen rotundum is posterior to the medial end of the superior orbital fissure and is where the maxillary branch of the trigeminal nerve (CN V2) travels. Posterolateral to the foramen rotundum is the foramen ovale, where the mandibular branch of trigeminal nerve (CN V3) exits. Further posterolateral from the foramen ovale is the foramen spinosum, which connects the middle cranial fossa to the infratemporal fossa by allowing the middle meningeal blood vessels and the lesser petrosal nerve to pass through [1]. Directly posterior to the foramen ovale and medial to the foramen spinosum is where the carotid canal is found, which transmits the internal carotid artery and its peri-arterial sympathetic plexus. Medial to the carotid canal is the foramen lacerum, which is usually filled with cartilage to allow the greater petrosal nerve to pass over. Although variations to the middle cranial fossa have been reported, the overall frequency of variation is rare [2,3]. We report a rare case of the variant bilateral foramina in the middle cranial fossa.

## Case presentation

**Figure 1:**
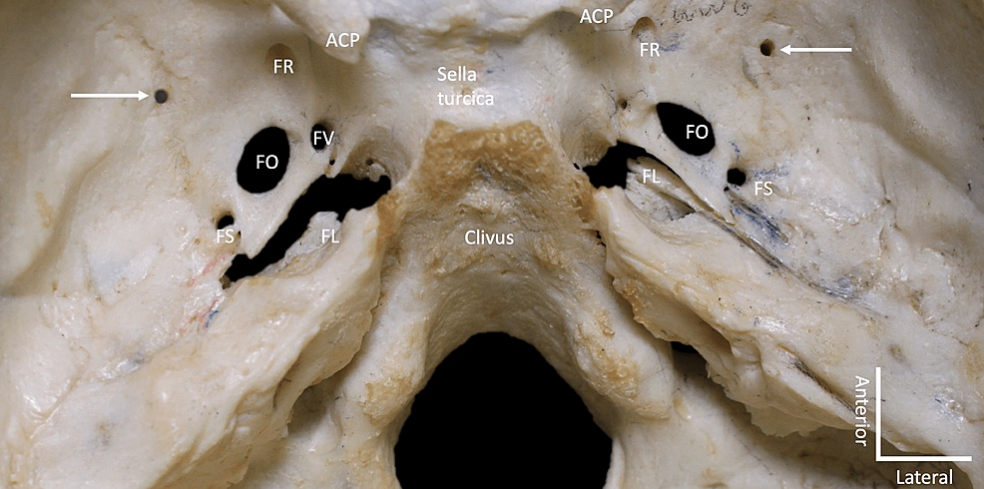
Superior view of the internal aspect of the skull base showing mostly the middle cranial fossa. Note the two variant foramina at the arrows. Also note the anterior clinoid process (ACP) foramen rotundum (FR), foramen ovale (FO), foramen spinosum (FS), foramen lacerum (FL), and foramen of Vesalius (FV).

**Figure 2:**
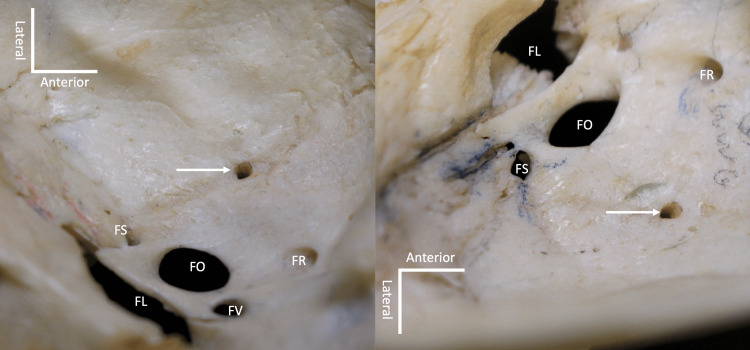
Magnified views of the variant foramina (arrows) in Figure 1. The left image showing the left variant foramen (medial view) and the right image showing the right variant foramen (lateral view). Note the foramen ovale (FO), foramen spinosum (FS), foramen lacerum (FL), foramen of Vesalius (FV), and foramen rotundum (FR).

**Figure 3:**
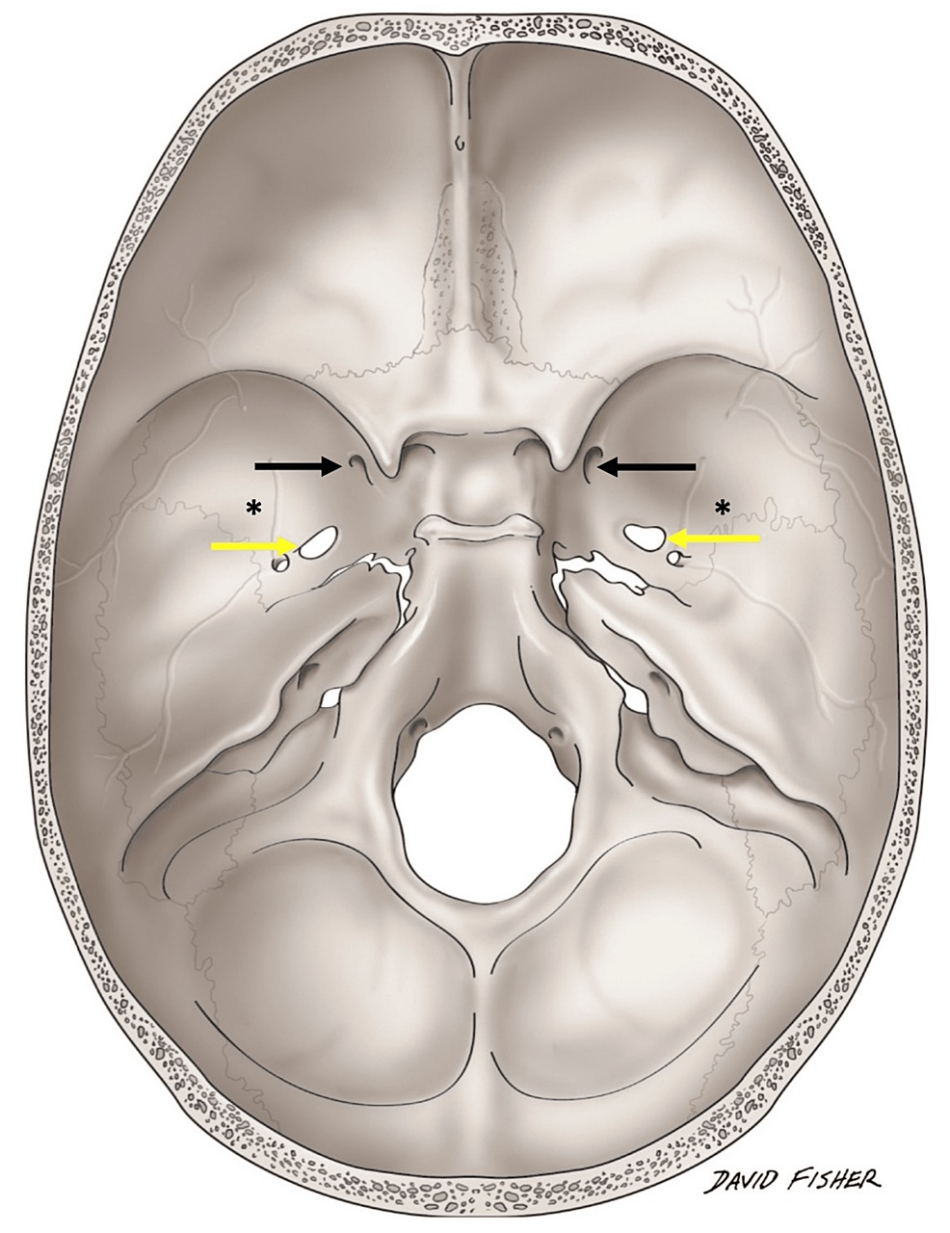
Schematic drawing of the internal aspect of the skull base. The foramina rotundum (black arrows) and foramina ovale (yellow arrows) are shown and their approximate relationship to the variant foramina (*) noted in the case presented here. Illustration courtesy of Mr. David Fisher.

We report an adult male skull found to have unusual foramina located at the skull base. This skull specimen was from a skeleton curated in our university’s osteological collection. The exact age was unknown but was estimated to be 50-60 years old. The variant foramen was observed bilaterally in the middle cranial fossa. The foramina were located approximately 3.1 cm lateral to the plane of the foramen rotundum and foramen ovale (Figures 1, 2, 3).

**Figure 4:**
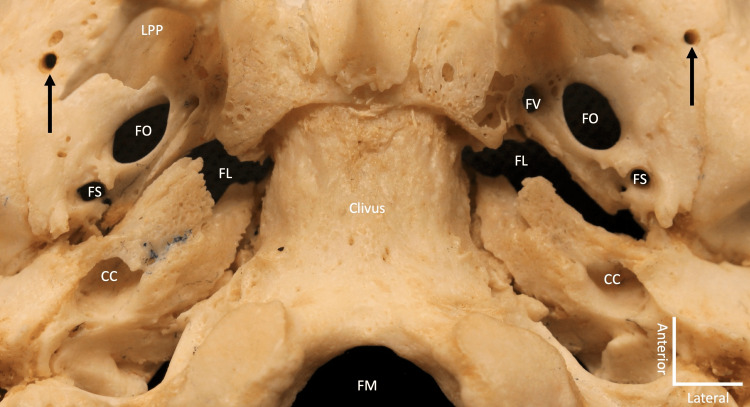
Inferior view of the external surface of the skull base noting the variant foramina (black arrows) seen in Figure 1. The opening of the variant foramen is adjacent to multiple small openings that enter the diploic space but not the middle cranial fossa. Note the carotid canal entrance (CC), lateral plate of the pterygoid process (LPP), foramen ovale (FO), foramen spinosum (FS), foramen lacerum (FL), and foramen magnum (FM).

**Figure 5:**
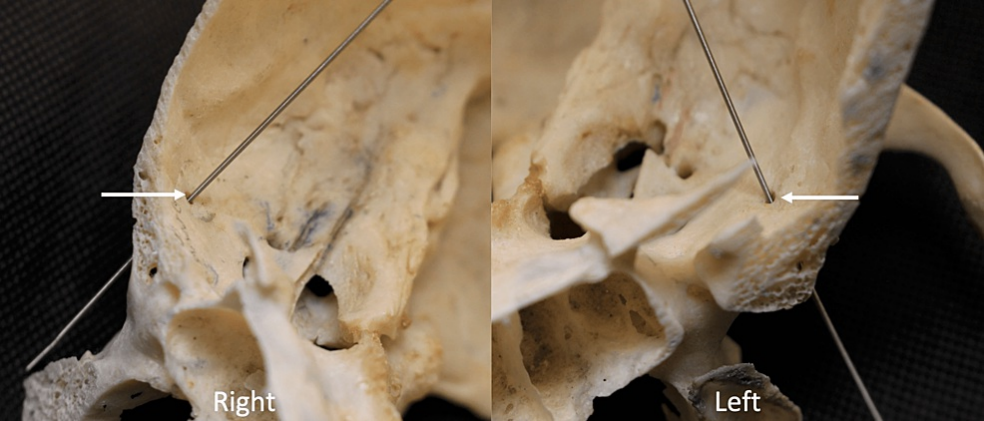
A probe was inserted into the foramen on each side. Note the skull was cut coronally through the sphenoidal sinus to show the foramina with probes (arrows).

The left foramen had a diameter of 0.82 mm and the right foramen had a diameter of 0.77 mm. Both foramina opened up just medial to the sphenotemporal suture into the roof of the infratemporal fossa (Figures 4, 5).

Additionally, each foramen was the most lateral of a larger collection of foramina found to exit the skull base but confluent with the diploic space of the greater wing of the sphenoid and not with the floor of the middle cranial fossa. This group of openings, including the most lateral which communicated with the middle cranial fossa, were lateral to the lateral plate of the pterygoid process. Other than a left-sided foramen of Vesalius, no other gross anatomical variations were noted in this skull specimen. Measurements were made with microcalipers (Mitutoyo, Kawasaki, Japan). 

## Discussion

We identified two laterally placed foramina in the floor of the middle cranial fossa. To our knowledge, such foramina have not been previously reported. Therefore, such information is of archival value when comparing any future similar findings. Based on the relationship of these variant foramina with other openings at the extracranial surface of the skull base, we would speculate that they carried emissary veins that connected the veins of the middle cranial fossa floor e.g., anterior temporal veins to the pterygoid venous plexus. 

Several variations to the middle cranial fossa have been documented, although morphological analysis of cadaveric skull bases has shown the overall frequency of variation to be rare. The major foramina that allow passage of the cranial nerves and carotid blood vessels consistently appear in virtually every morphological study of the middle cranial fossa [2]. Variations of the anatomically consistent foramina of the middle cranial fossa mainly involve the foramen ovale and foramen spinosum [3]. Aside from the variations of the consistent foramina, the appearance of accessory foramina medial to the foramen ovale and foramen spinosum are relatively common variations found in the middle cranial fossa. Reports of variant foramina located lateral to the foramen spinosum and foramen ovale are difficult to identify in the extant medical literature. 

The two most consistently identified accessory foramina of the middle cranial fossa are the foramen innominatus and foramen of Vesalius. Both variant foramina may present unilaterally or bilaterally [4]. The foramen innominatus, or canaliculus innominatus, is found between the foramen ovale and foramen spinosum. This passage allows the lesser petrosal nerve to pass through alone as opposed to traveling with the middle meningeal vessels in the foramen spinosum [5]. The foramen of Vesalius is anteromedial to the foramen ovale. It is also referred to as the emissary sphenoidal foramen because it carries a vein that connects the pterygoid venous plexus with the cavernous sinus. The opening created by the foramen of Vesalius could play a significant pathophysiological role in infections that enter the cavernous sinus extracranially [6]. Surgical procedures for trigeminal neuralgia targeting the foramen ovale must have careful consideration regarding the presence of accessory foramina such as the foramen of Vesalius. And the variant foramina described in the present case. Unintentionally puncturing such foramina and their emissary veins during percutaneous intervention could cause intracranial bleeding [7]. Embryologically, the passages for the cranial nerves are formed by the 12th week of fetal development. So the present anomaly should be formed before it as ossification might have occurred around nerve branches or vessels [8]. Therefore, anatomical consideration of the accessory foramina could reduce the risk of surgical complications in percutaneous approaches to the middle cranial fossa through the foramen ovale.

## Conclusions

This group of openings, including the most lateral which communicated with the middle cranial fossa, were lateral to the lateral plate of the pterygoid process. Knowledge of variant foramina of the skull base is important to clinicians treating patients with pathology of this region. As the case presented here appears to be extremely rare, it is of significant archival value.
